# Evaluation of the Antimicrobial Potential and Characterization of Novel T7-Like *Erwinia* Bacteriophages

**DOI:** 10.3390/biology12020180

**Published:** 2023-01-23

**Authors:** Su Jin Jo, Sang Guen Kim, Young Min Lee, Sib Sankar Giri, Jeong Woo Kang, Sung Bin Lee, Won Joon Jung, Mae Hyun Hwang, Jaehong Park, Chi Cheng, Eunjung Roh, Se Chang Park

**Affiliations:** 1 Laboratory of Aquatic Biomedicine, College of Veterinary Medicine and Research Institute for Veterinary Science, Seoul National University, Seoul 08826, Republic of Korea; 2 Laboratory of Aquatic Nutrition and Ecology, College of Animal Science and Technology, Nanjing Agricultural University, Nanjing 210095, China; 3 Crop Protection Division, National Institute of Agriculture Sciences, Rural Development Administration, Wanju 55365, Republic of Korea

**Keywords:** Bacteriophage, *Erwinia* blight, pome fruit, phage cocktail, agriculture

## Abstract

**Simple Summary:**

*Erwinia amylovora* and *E. pyrifoliae* cause *Erwinia* blight, which damage pome fruits, and are highly contagious. We propose the use of bacteriophages to control these two pathogens simultaneously. Many drugs have been used in South Korea for the quick control of blight disease caused by both these species. This can result in antibiotic resistance; hence, phage cocktails have been suggested as an alternative. In this study, we observed that phage cocktails, including four isolated phages, exhibited extensive strain spectra and potential for rapid bacterial control. This study demonstrated the potential of a phage cocktail to replace antibiotics as biocontrol agents against *Erwinia* blight.

**Abstract:**

The recent outbreak of blight in pome fruit plants has been a major concern as there are two indistinguishable *Erwinia* species, *Erwinia amylovora* and *E. pyrifoliae*, which cause blight in South Korea. Although there is a strict management protocol consisting of antibiotic-based prevention, the area and the number of cases of outbreaks have increased. In this study, we isolated four bacteriophages, pEp_SNUABM_03, 04, 11, and 12, that infect both *E. amylovora* and *E. pyrifoliae* and evaluated their potential as antimicrobial agents for administration against *Erwinia*-originated blight in South Korea. Morphological analysis revealed that all phages had podovirus-like capsids. The phage cocktail showed a broad spectrum of infectivity, infecting 98.91% of *E. amylovora* and 100% of *E. pyrifoliae* strains. The antibacterial effect was observed after long-term cocktail treatment against *E. amylovora*, whereas it was observed for both short- and long-term treatments against *E. pyrifoliae*. Genomic analysis verified that the phages did not encode harmful genes such as antibiotic resistance or virulence genes. All phages were stable under general orchard conditions. Collectively, we provided basic data on the potential of phages as biocontrol agents that target both *E. amylovora* and *E. pyrifoliae.*

## 1. Introduction

A major pathogenic bacterium of the pome fruit plant, *Erwinia amylovora*, has recently been introduced into South Korea [1,2,3,4]. *E. amylovora* has been reported to result in symptoms indistinguishable from those of *E. pyrifoliae*, an endemic pathogen in South Korea [5,6,7,8]. Both pathogens cause blight disease with the blackening of leaves, stems, and immature fruits, starting with flower infection [9,10,11,12,13]. As *E. amylovora* is regulated by law, the disease management protocol should be performed in a different way compared to *E. pyrifoliae* outbreaks [14]. Therefore, strict regulations are applied to *E. amylovora* outbreaks, with orchards being forcibly closed at 5% outbreak rates (or less) at the discretion of the government plant-disease control agent [15,16].

Periodic surveillance and prevention-based disease control programs must be performed to prevent the spread of these two pathogens [17]. The general protocol for fire blight prevention consists of three antibiotic administrations (once before flowering and twice during the flowering period). To prevent black shoot blight, antibiotics are administered twice after full bloom [15,18]. Despite the intensive disease control program and antibiotics for *Erwinia*-associated blight, the outbreak of fire blight has been on the rise, with an increased possibility of the evolution of antibiotic resistance among pathogenic strains [19,20]. Therefore, it is necessary to develop more effective agents other than antibiotics for the treatment of pathogenic *Erwinia* species [21,22,23].

Bacteriophages (phages) have been used as effective antimicrobial agents for the treatment of fire blight worldwide [24,25,26]. Phages are “smart biocontrol agents” as they replicate at the targeted infection site, enabling prolonged antimicrobial effects on-site [27,28]. The infection specificity of phages allows specific pathogens to be targeted while maintaining beneficial microbes in the environment [29,30]. To maximize the antimicrobial effects of phages, a combination of phages with different host spectra is used to exert antimicrobial effects over a wider range of pathogens; this pret-a-porter approach is one of the main paradigms for therapeutic phage preparation [31,32,33]. Furthermore, cocktail phage therapy, which is a combinatorial strategy, has been reported to have a synergistic effect [34,35,36,37,38].

This study investigated the biological control potential of the newly isolated *Erwinia* phages. The biological and genomic characteristics, including morphology, stability, and antimicrobial potential of four phages that showed infectivity toward both *E. amylvoroa* and *E. pyrifoliae* were examined in this study.

## 2. Materials and Methods

### 2.1. Phage Isolation

Water and soil samples were collected near the location where the blight outbreak occurred in South Korea to isolate phages that infect *E. pyrifoliae*. Phages were isolated as previously described [39]. Distilled water (10 mL) was added to the soil samples (1g). The samples were centrifuged at 10,000× *g* for 10 min to remove contaminants. A host strain suspension (1%, *v*/*v*) containing *E. amylovora* (TS3128) or *E. pyrifoliae* (KACC13945) was cultured overnight for approximately 18 h at 27 °C. The suspension was used to inoculate the samples and nutrient broth (NB; Difco) for phage enrichment and cultured for 24 h at 27 °C. After enrichment, serial dilutions of the culture broth were transferred onto bacterial lawns of the *E. amylovora* (TS3128) or *E. pyrifoliae* (KACC13945). Phage isolation was confirmed using a double-layer agar assay. The double-layer agar assay was used to verify bacteriolysis induced by the inhibition spots of phages. The samples showing plaque formation were centrifuged at 10,000× *g* and passed through 0.45 μm syringe filters. Pure phages were obtained by picking a single plaque and subjecting it to a double layer assay five times.

### 2.2. Phage Propagation and Purification

Phage propagation was conducted as previously described [40]. The overnight culture (1%) was inoculated with different multiplicity of infection (MOIs; 10, 5, 1 and 0.1) of phages to determine the optimum ratio for phage propagation and cultured for 24 h at 27 °C. Phage lysate was centrifuged at 12,000× *g* for 10 min and the supernatant was precipitated with 10% (*w*/*v*) polyethylene glycol/ 0.5 M NaCl. (final concentration). A cesium chloride (CsCl) gradient was used to purify the phage suspension [41]. The gradient layers were ultracentrifuged at 182,000× *g* for 3 h. Phage precipitation bands were collected and dialyzed using a dialysis bag (Slide-A-Lyzer™ Dialysis Cassettes, 10,000 MWCO).

### 2.3. Transmission Electron Microscopy (TEM)

Purified phage suspensions (10 μL) were mixed with the same volume of uranyl acetate (2%). The suspensions were incubated on a copper grid for 1 min. The excess sample was removed and washed with distilled water. Images of the phages were obtained using a Talos L120C (FEI, Hillsboro, OR, USA) at 120 kV. The dimensions of four independent phages were determined (*n* = 5).

### 2.4. Host Range

All the bacterial strains used in the host range assay were recent isolates from the blight tissues in South Korea. A total of 116 bacterial strains, including 92 *E. amylovora* and 24 *E. pyrifoliae* strains were spot assayed on nutrient agar (NA; Difco) plates with serial dilutions (10^−1^ to 10^−8^) of purified phage suspension; the plates were incubated for 24 h at 27 °C [40]. Plaque formation on the spot areas resulted in the bacterial strain being considered susceptible and is represented as “+” in Table S1. The experiments were performed in triplicates.

### 2.5. Stability Test

The thermal stability of the phages was evaluated as described by Kim et al. [42]. Phage suspensions (1 mL each, 2 × 10^8^ PFU/mL) were incubated for 60 min at 4 (control), 20, 30, 40, and 50 °C. Approximately 100 μL aliquots of each suspension were used to determine the concentration of phages using a double-layer agar assay. The pH stability of the phages was evaluated by adjusting the pH of phage suspensions (2 × 10^8^ PFU/mL) to 4.0, 5.0, 6.0, 7.0 (control), 8.0, and 9.0 with 0.1 M HCl and 0.1 M NaOH; each of the phage suspensions was then incubated for 60 min at 27 °C. They were then evaluated using a double-layer agar assay. All tests were performed in triplicate.

### 2.6. One-Step Growth Curve

The phage suspension (100 μL) was inoculated onto 10 mL of exponentially growing host strain culture (2 × 10^8^ colony-forming units [CFU]/mL) at an MOI of 0.001 [43]. The phages were allowed to infect the bacterial cells for 10 min and the suspension was centrifuged at 12,000× *g* to remove unattached phages. The phage-infected bacterial pellets were then resuspended in preheated NB (10 mL) and incubated at 27 °C with shaking (150 rpm). Aliquots (100 μL) were collected at 5 min intervals for 50 min; the titers were then evaluated using a double-layer agar assay. The experiments were performed in triplicate. 

### 2.7. Genome Analysis

Genomic DNA was extracted from phages as described previously [34,39]. Purified phage suspension (≥10^10^ PFU/mL) was digested with 10 IU of DNase I and RNase A to remove nucleotides originating from the hosts. The nucleases were heat-inactivated at 95 °C by the addition of EDTA. Proteinase K and SDS (10%) were added to the samples to degrade structural proteins. DNA was purified with phenol-chloroform-isopropanol and precipitated with absolute ethanol, followed by two washes with 70% ethanol. The phage genomic DNA was sequenced using an Illumina HiSeq platform at Macrogen (Seoul, South Korea). The short reads were assembled into contigs using de bruin graphs in CLC genomic workbench (v. 6.5.1). Open reading frames (ORFs) were identified using GenMarkS and Rapid Annotation using subsystem Technology (RAST) [44,45]. The presence of tRNA, and virulence and antibiotic genes was determined using tRNAscan-SE, VirulenceFinder, and ResFinder, respectively [46,47,48]. Comparative genome analysis was performed based on sequence similarity using tBLASTx [49]. Whole-genome phylogenetic analysis was performed using the Virus Classification and Tree Building Online Resource (VICTOR) with the recommended setting for complete nucleotide sequences [50].

### 2.8. Antibacterial Activity

The antibacterial effect of pEp_SNUABM_03, 04, 11, and 12 was evaluated over short (2 h) and long (8 h) periods of time. The assay was performed using two indicator strains, *E. amylovora* (TS3128) and *E. pyrifoliae* (KACC13945). The phage cocktail was prepared by combining the four phages at equal ratios (1:1:1:1) to obtain 2 × 10^8^ PFU/mL. Exponentially growing indicator strains were inoculated into fresh NB to obtain 2 × 10^5^ CFU/mL for 8 h and at 27 °C, and the phage suspension was inoculated into the broth at three concentrations (MOI 5, 1, and 0.1). The mixtures were cultured with shaking at 150 rpm, and CFUs were determined. The CFU values were determined by preparing serial dilutions in phosphate buffered saline and plating for the quantification of viable bacteria. All tests were performed in triplicate.

### 2.9. Statistical Analysis

Statistical differences were analyzed using Sigmaplot 12.5 (Systat Software Inc., Evanston, IL, USA) using analysis of variance with the Holm–Sidak test. Statistical significance was set at *p* < 0.05.

## 3. Results

### 3.1. TEM—Biological Analysis

**Figure 1:**
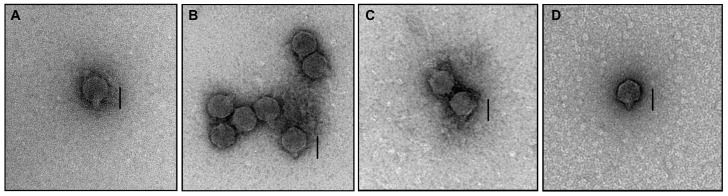
Morphological observation by transmission electron micrographs of *Erwinia pyrifoliae* phages (**A**) pEp_SNUABM_03, (**B**) pEp_SNUABM_04, (**C**) pEp_SNUABM_11, and (**D**) pEp_SNUABM_12. Scale bar = 50 nm.

Morphological observations using TEM revealed four distinct phages that belong to *Podoviridae* (Figure 1). Structural observations of pEp_SNUABM_03, 04, 11, and 12 revealed short tails with head diameters of 56 ± 2, 55 ± 3, 56 ± 3, and 63 ± 2 nm (*n* = 5), respectively (Table 1).

### 3.2. Stability Test

**Figure 2:**
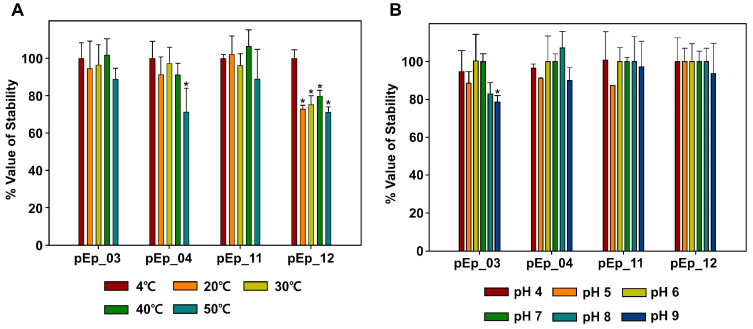
Stability of phages pEp_SNUABM_03, pEp_SNUABM_04, pEp_SNUABM_11, and pEp_SNUABM_12 at thermal (**A**) and pH (**B**) stresses. Phages were incubated for 1 h under each condition and the phage titer was determined on the host strain. One-way ANOVA with Holm–Sidak tests were performed to determine significant differences (*p* < 0.05; *n* = 3).

The test was conducted under normal-orchard environmental temperature and pH conditions (Figure 2). Thermal stability tests showed that pEp_SNUABM_03 and 11 were stable at 4 (control), 20, 30, 40, and 50 °C for 1 h, and virions of pEp_SNUABM_04 were vulnerable to high temperature (50 °C; *P* < 0.05). The phage pEp_SNUABM_12 was sensitive to temperature changes (*P* < 0.05). The pH stability test revealed that pEp_SNUABM_04, 11, and 12 were all stable, whereas the stability of pEp_SNUABM_03 decreased at pH 9 (*P* < 0.05).

### 3.3. One-Step Growth Curve

**Figure 3:**
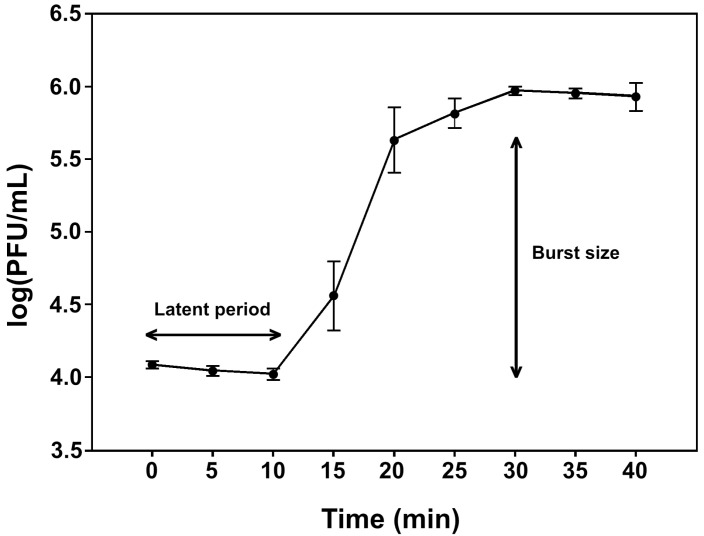
One-step growth curve of the pEp_SNUABM_03 in *E. pyrifoliae* strain KACC13945. The values are presented as mean ± standard deviation.

All four phages exhibited similar biological characteristics. Hence pEp_SNUABM_03 was used as a representative phage for one-step growth analysis (Figure 3). After the 10-min latent period, the first burst size of the phage growth was 76.83 PFU per bacterial cell for pEp_SNUABM_03.

### 3.4. Genome Analysis

**Figure 4:**
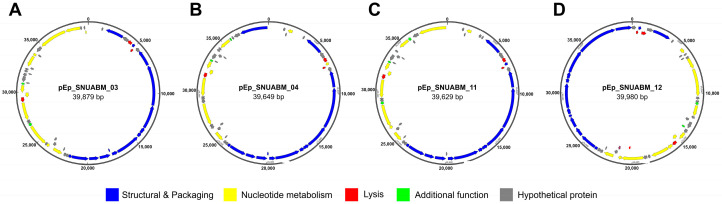
Genome map of *Erwinia* phages (**A**) pEp_SNUABM_03, (**B**) pEp_SNUABM_04, (**C**) pEp_SNUABM_ 11, and (**D**) pEp_SNUABM_12 The color-coded ORFs are classified based on their function (Scale = base pair).

The general characteristics of phages pEp_SNUABM_03, pEp_SNUABM_04, pEp_SNUABM_11, and pEp_SNUABM_12 are listed in Table 2. A total number of reads 3,864,800 (pEp_SNUABM_03), 3,730,842 (pEp_SNUABM_04), 3,426,138 (pEp_SNUABM_11), 3,818,762 (pEp_SNUABM_12) were obtained from the Illumina sequencer, which was assembled into the single contig. The circular genomes of phages pEp_SNUABM_03, pEp_SNUABM_04, pEp_SNUABM_11, and pEp_SNUABM_12 contained 39,879, 39,649, 39,626, and 39,980 bp with GC contents of 52.13%, 52.19%, 52.10%, and 51.19%, respectively (Table 2). A total of 52, 52, 49, and 50 ORFs were identified in the genomes of pEp_SNUABM_03, pEp_SNUABM_04, pEp_SNUABM_11, and pEp_SNUABM_12, respectively. The function of the predicted ORFs was categorized into five groups: structural and packaging proteins, nucleotide metabolism-related proteins, lysis proteins, additional function proteins, and hypothetical proteins (Figure 4).

**Figure 5:**
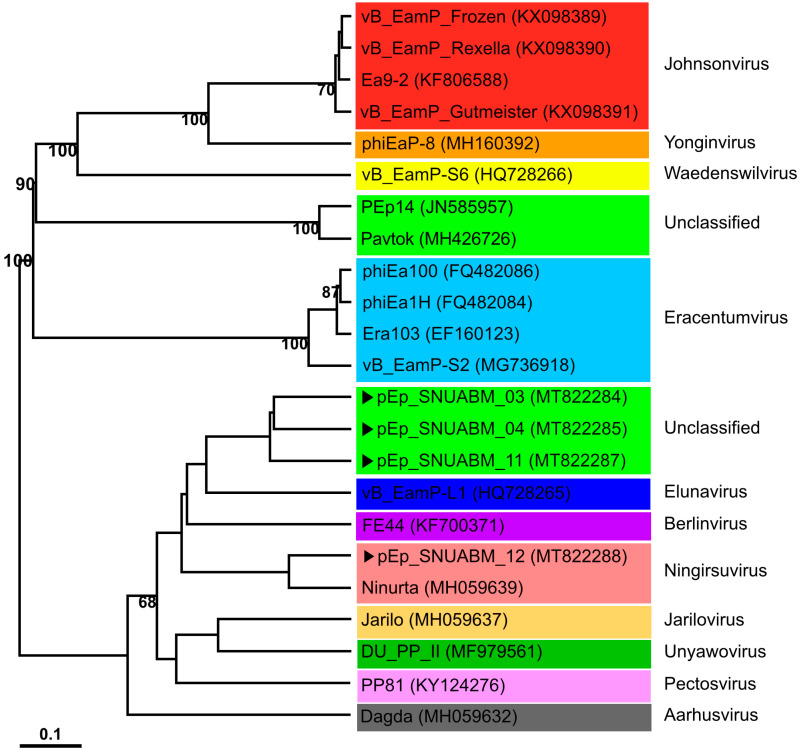
Whole-genome phylogenetic analysis of newly isolated *Erwinia* phages. The four phages isolated in this study are indicated with arrows (▶). The different genera (*Johnsonvirus*, red box; *Yonginvirus*, orange box, *Waedenswilvirus*, yellow box; unclassified, light green; *Eracentumvirus*, sky-blue box; *Elunavirus*, deep blue box; *Berlinvirus*, purple box; *Ningsuvirus*, pink box; *Jarilovirus*, light orange box; *Unyawovirus*, green box; *Pectosvirus*, purple box and *Aarhusvirus*, gray box) are indicated using colors.

The phylogenetic positions of phages pEp_SNUABM_03, pEp_SNUABM_04, pEp_SNUABM_11, and pEp_SNUABM_12, which have the morphology of podovirus, were analyzed using the complete genome sequences of closely related phages infecting *Enterobacterales* (*Erwinia*, *Dickeya*, and *Pectobacterium*). All phages were classified under the subfamily *Studiervirinae* in the family *Autographiviridae* (Figure 5). Phage pEp_SNUABM_12 clustered with *Ningirsuvirus* and the dickey phage Ninurta, whereas the other three phages were unclassified. Phages pEp_SNUABM_03, 04, and 11 were clustered with *Erwinia* phage vB_EamP-L1 belonging to *Elunavirus*. This cluster was most closely related to FE 44, another *Erwinia* phage belonging to *Berlinvirus*. Two clusters of the newly isolated phages branched from a common ancestor.

**Figure 6:**
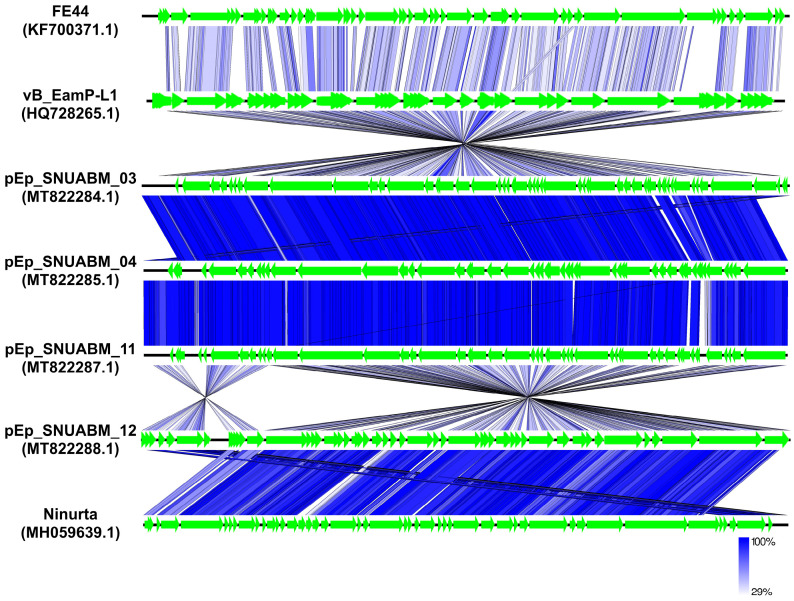
Comparative whole-genome analysis of *Erwinia* phages pEp_SNUABM_03, pEp_SNUABM_04, pEp_SNUABM_11, and pEp_SNUABM_12 among phages infecting *Enterobacterales* species. The tBLASTx comparison analysis was constructed with tBLASTx algorithm using Easyfig.

Comparative genome analysis supported the genomic distance between phages in the two clusters. The genomes of three unclassified phages, pEp_SNUABM_03, 04, and 11, showed highly conserved synteny revealing around 98% of nucleotide identity among them (thick blue), whereas the similarity level was low (nucleotide identity: around 70%; pale blue) with the closest neighbor, vB_EamP_L1 (Figure 6; Table S2). Phage pEp_SNUABM_12 showed high synteny with Ninurta (nucleotide identity: 94.66%), another member of *Ningirsuvirus* (Figure 6; Table S2) and genetic distance with pEp_SNUABM_03, 04, and 11. The three unclassified *Autographiviridae* phages shared more than 47 core genes, which accounted for more than 90% of their genes (Table S3). The shared genes among the four phages isolated in this study decreased to only 37 genes, as revealed by the comparative blast analysis (Tables S4–S7).

### 3.5. Host Range

Host range analysis was performed against 116 *Erwinia* strains including 92 *Erwinia amylovora* and 24 *Erwinia pyrifoliae* (Table 3). pEp_SNUABM_03 and 04 showed broad-host-spectrum infectivity to both *E. amylovora* (98.91%, 91/92; 97.83%, 90/92) and *E. pyrifoliae* (91.67%, 22/24; 95.83%, 23/24) strains, respectively. Although pEp_SNUABM_11 had a relatively narrow host range compared to pEp_SNUABM_03 and 04, it was highly infective (*E. amylovora*: 76.09%, 70/92; *E. pyrifoliae*: 79.17%, 19/24). Phage pEp_SNUABM_12 showed specific infectivity in *E. pyrifoliae* (95.83%, 23/24). pEp_SNUABM_12 was able to infect only two *E. amylovora* strains (2.17%, 2/92). The phage cocktail infected almost all *E. amylovora* (98.91%, 91/92) and *E. pyrifoliae* (100%, 24/24) strains.

### 3.6. Antibacterial Activity of Phages on E. amylovora

**Figure 7:**
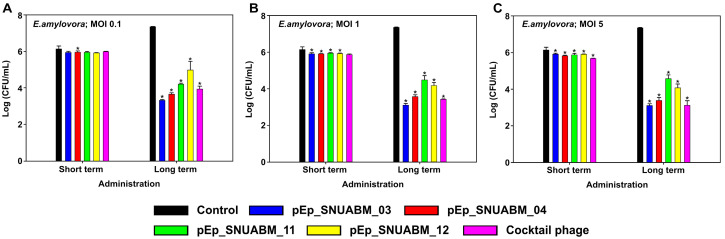
Evaluation of antibacterial activity of phages on *Erwinia amylovora*. The assay was performed at an MOI of 0.1 (**A**), 1 (**B**), and 5 (**C**). Statistical significance was calculated using a one-way analysis of variance (ANOVA) with Holm-Sidak tests (*p* < 0.001).

The antibacterial efficacy of the newly isolated phages was evaluated at three concentrations (MOI 0.1, 1, and 5) over short (2 h) and long (8 h) time periods (Figure 7). Phages pEp_SNUABM_03, 04, 11 and 12 co-cultured with *E. amylovora* TS3128 at an MOI of 0.1 resulted in a slight inhibition of bacterial growth in the short term; pEp_SNUABM_04 showed significant inhibition after administration (*p* < 0.05). In the long term, the antibacterial effect was significant for all phages (*p* < 0.001), pEp_SNUABM_03 (−4.03 logCFU/mL), 04 (−3.70 logCFU/mL), 11 (−3.14 logCFU/mL), and 12 (−2.37 logCFU/mL). At an MOI of 1, all phages showed a significant inhibitory effect against TS3128 after short-term administration (*p* < 0.05). In the long term, all phages showed a significantly increased antibacterial effect, pEp_SNUABM_03 (−4.24 logCFU/mL), 04 (−3.78 logCFU/mL), 11 (−2.86 logCFU/mL), and 12 (−3.18 logCFU/mL) (*p* < 0.001). Phages pEp_SNUABM_03, 04, 11 and 12, were co-cultured with TS3128 at an MOI of 5 and exhibited significant inhibition of bacterial growth in the short term for all phages (*p* < 0.05). In the long term, there were notable reductions in bacterial counts for all phages; pEp_SNUABM_03 (−4.24 logCFU/mL), 04 (−3.97 logCFU/mL), 11 (−2.77 logCFU/mL), and 12 (−3.29 logCFU/mL) (*p* < 0.001).

The phage cocktail consisted of an equal ratio of the four phages, resulting in the same overall concentration as solely administered phages. Although one-fourth of each of the phages were combined, the antibacterial effect of the cocktail phage suspension administered over the long term, −3.42 logCFU/mL (MOI 0.1), −3.93 logCFU/mL(MOI 1), and −4.23 logCFU/mL (MOI 5), was higher than the average CFU reduction exhibited by individual phages, which is indicative of a synergistic effect.

### 3.7. Antibacterial Activity of Phages on E. pyrifoliae

**Figure 8:**
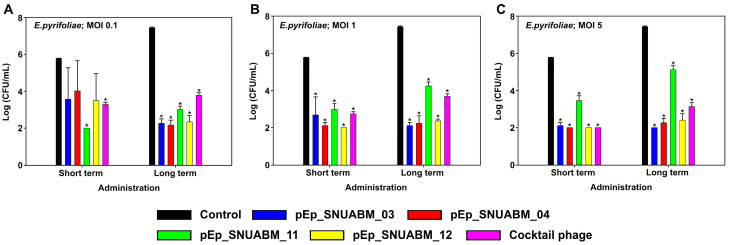
Evaluation of antibacterial activity of phages on *Erwinia pyrifoliae.* The assay was performed at an MOI of 0.1 (**A**), 1 (**B**), and 5 (**C**). Statistical significance was determined using a one-way analysis of variance (ANOVA) with Holm-Sidak tests (*p* < 0.001).

The antibacterial effects of the four phages were evaluated at three concentrations (MOI 0.1, 1, and 5) over short (2 h) and long (8 h) periods of time (Figure 8). All phages showed rapid antibacterial effects against *E. pyrifoliae*. When *E. pyrifoliae* KACC13945 and phages pEp_SNUABM_03, 04, 11, and 12 were co-cultured at an MOI of 0.1, bacterial growth was inhibited in the short term, with pEp_SNUABM_11 showing significant inhibition (*p* < 0.05). In the long term, the antibacterial effect significantly decreased for all phages (*p* < 0.001), pEp_SNUABM_03 (−5.17 logCFU/mL), 04 (−5.27 logCFU/mL), 11 (−4.43 logCFU/mL), and 12 (−5.10 logCFU/mL). At an MOI of 1, all phages rapidly inhibited bacterial growth after short-term administration and showed a significant inhibitory effect against KACC13945 (*p* < 0.001). In the long term, the antibacterial effect was sustained in all phages; pEp_SNUABM_03 (−5.33 logCFU/mL), 04 (−5.20 logCFU/mL), 11 (−3.19 logCFU/mL), and 12 (−5.07 logCFU/mL) (*p* < 0.001). Phages pEp_SNUABM_03, 04, 11, and 12 co-cultured with KACC13945 at an MOI of 5 showed considerable reductions in bacterial counts in the short term for all phages (*p* < 0.001). In the long term, the antibacterial effect was maintained, and the bacterial counts were significantly reduced for all phages (*p* < 0.001); pEp_SNUABM_03 (−5.43 logCFU/mL), 04 (−5.17 logCFU/mL), 11 (−2.31 logCFU/mL), and 12 (−5.03 logCFU/mL).

The antibacterial efficacy of the phage cocktail suspension administered over a short term was −2.49 logCFU/mL (MOI 0.1), −3.03 logCFU/mL (MOI 1), and −3.77 logCFU/mL (MOI 5). Whereas the average CFU reduction in each phage, −2.50 logCFU/mL (MOI 0.1), −3.15 logCFU/mL (MOI 1), and −3.38 logCFU/mL (MOI 5), did not exhibit any synergy effect of the cocktail phage. However, there was a significant decrease in the bacterial count in the short-term phage cocktail treatment.

## 4. Discussion

*Erwinia*-associated blight disease in rosaceous fruit plants in South Korea is caused by *E. pyrifoliae* infection [6]. However, the recent outbreak of fire blight caused by *E. amylovora* has rendered the disease management protocol complicated, as a co-outbreak with *E. pyrifoliae* was identified [5,51]. In contrast to *E. pyrifoliae*, fire blight caused by *E. amylovora* is registered as a legal communicable disease in plants in South Korea, and there is a distinct disease management protocol [13,16,52]. To provide an effective control method against both pathogens, we isolated and characterized the potential of bacteriophages against *Erwinia*-originated blight disease in South Korea.

The rosaceous fruit plant industry has tried to use phages as biocontrol agents against *E. amylovora* outbreaks worldwide [53,54]. A number of phages have been isolated, and their potential as antimicrobial agents has been confirmed [34,55,56]. A cocktail phage suspension that combines phages with different infection mechanisms is preferred over individual phage isolates to minimize resistance and maximize the antibacterial effect for effective disease control [34,57,58]. As *Erwinia* bacteriophages have a broad host range, the major objective of their combined administration is to improve their antimicrobial potential [36,59]. The four phages used in this study also had a broad host range, except for pEp_SNUABM_12, which specifically infects *E. pyrifoliae* (Table 3). Phages use distinct infection strategies based on their tail structure, and the infectivity of the four phages is distinct from each other [60,61]. This suggests that they have different infection strategies that would prevent the prevalence of resistant bacterial strains [28,62].

Several studies have shown that phage resistance in bacterial strains is present in the form of a trade-off [63,64]; bacteria acquire phage resistance in return for fitness loss, including growth, virulence, and antibiotic susceptibility [65,66,67]. Attenuation or loss of virulence has been observed in several strains of *Pectobacterium atrosepticum* and *Pseudomonas plecoglossicida* resistant against phages PPpW-3 and/or PPpW-4, respectively [68,69]. Impaired growth characteristics have been reported in phage-resistant *E. amylovora* and *P. syringae*, which significantly affected their virulence [70,71]. Phage-resistant *Escherichia coli*, and *E. amylovora* strains become more susceptible to antibiotics [34,72]. Furthermore, *E. amylovora* bacteriophages showed transient resistance in infected bacterial strains, with phage infectivity being restored after the phage was eliminated.

Synergism is one of the major incentives for combining several phages in a cocktail suspension [36,37]. A synergistic effect refers to the antimicrobial potential of cocktail phages being greater than the sum of the individual phages; an additive effect occurs when a cocktail phage provides the sum of the effects of individual phages; an antagonistic effect refers to the antimicrobial potential of the cocktail phages being less than that of the sum of the individual phages [73]. The best selection for phage cocktail components results in synergy; as observed in our study (Figure 7), there should be no antagonistic effect between the cocktail phages. As phages can interrupt secondary infections by closely related phages, it is recommended that antagonistic phages be excluded at the first selection step.

The stability of phages under environmental stress should be verified before their application. The major stress factors expected are acidity, temperature, and UV radiation [74]. Although increased stability of the phages better facilitates their application as biocontrol agents, there are several ways to bypass environmental stresses (Figure 2). Control agents can be administered in the morning or encapsulated to minimize exposure to temperature and light, or acidity, respectively [75,76].

Although the efficacy and stability of phages are guaranteed, safety is a major concern. Generally, phages with an obligatory lytic life cycle are preferred as biocontrol agents against *Erwinia*-originated blight diseases (Figure 4). On the other hand, lysogenic phages have a greater potential for transducing harmful genes including those associated with antimicrobial resistance, virulence, and toxins [77]. However, if the transduction issue is eliminated, lysogenic phages may also be good candidates for controlling fire blight [78].

In the present study, the efficacy of the four phages and the phage cocktail against *Erwinia* strains indicates its possible use as a biocontrol agent under field conditions. The antibacterial effect can be further improved through modifications in the cocktail ratio as the phages exhibited synergy. To be applied in the actual environment, future studies should focus on the biocontrol efficacy of optimum phage cocktails *in planta* and carry out acute ecotoxic tests in fish to rule out possible environmental health hazards.

## 5. Conclusions

We isolated four phages, pEp_SNUABM_03, 04, 11, and 12, effective against both *E. amylovora* and *E. pyrifoliae* pathogens, and investigated their biological and genomic properties. Phages showed infectivity to both pathogens of *Erwinia* and were able to control these pathogens effectively over a long period of time. The cocktail treatment has the advantage of broadening the host spectrum as well as inducing synergistic effects. In addition, the stability and safety of phages for use as biocontrol agents were verified. Taken together, combining several phages that have distinct infection strategies and administering the cocktail phage suspension would be a remarkable way to control both *Erwinia amylovora* and *E. pyrifoliae* caused blight disease in South Korea. However, intensive verifications such as combined treatment with conventional agents, antibacterial efficacy *in planta*, and field tests, should be performed in further studies.

## Figures and Tables

**Table 1:** Morphological characteristics of *Erwinia* phages.

Phage	Capsid (nm)	Tail Length (nm)	Virus Family
pEp_SNUABM_03	56 ± 2	17 ± 2	*Podoviridae*
pEp_SNUABM_04	55 ± 3	16 ± 2	*Podoviridae*
pEp_SNUABM_11	56 ± 3	18 ± 1	*Podoviridae*
pEp_SNUABM_12	63 ± 2	17 ± 1	*Podoviridae*

**Table 2:** General genomic features of *Erwinia* phages.

Phage	Genome Size (bp)	ORFs	GC Content (%)	DNA Circularity	Accession Number
pEp_SNUABM_03	39,879	52	52.13%	circular	MT822284.1
pEp_SNUABM_04	39,649	52	52.19%	circular	MT822285.1
pEp_SNUABM_11	39,626	49	52.10%	circular	MT822287.1
pEp_SNUABM_12	39,980	50	51.19%	circular	MT822288.1

**Table 3:** Host range analysis of individual and combined *Erwinia* phages, alone and as and the combined cocktail.

Bacteria	pEp_SNUABM_03	pEp_SNUABM_04	pEp_SNUABM_11	pEp_SNUABM_12	Cocktail Phage
*E. amylovora*	98.91% (91/92)	97.83% (90/92)	76.09% (70/92)	2.17% (2/92)	98.91% (91/92)
*E. pyrifoliae*	92.00% (22/24)	95.83% (23/24)	79.17% (19/24)	95.83% (23/24)	100.00% (24/24)

## Data Availability

The genome sequences of the four *Erwinia* phages (pEp_SNUABM_03, pEp_SNUABM_04, pEp_SNUABM_11, and pEp_SNUABM_12) were deposited at GenBank under the accession numbers MT822284.1, MT822285.1, MT822287.1, and MT822288.1, respectively.

## Supplementary Materials

The following supporting information can be downloaded at: https://www.mdpi.com/article/10.3390/biology12020180/s1, Table S1: Host range of phage pEp_SNUABM_03, pEp_SNUABM_04, pEp_SNUABM_11, pEp_SNUABM_12, and Cocktail phage; Table S2: Nucleotide identity (%) among the closely related phages; Table S3: Coregene of *Erwinia* phages pEp_SNUABM_03, pEp_SNUABM_04, pEp_SNUABM_11, pEp_SNUABM_12; Table S4: Functional classification of ORFs in *Erwinia* phage pEp_SNUABM_03; Table S5: Functional classification of ORFs in *Erwinia* phage pEp_SNUABM_04; Table S6: Functional classification of ORFs in *Erwinia* phage pEp_SNUABM_11 Table S7: Functional classification of ORFs in *Erwinia* phage pEp_SNUABM_12.
