# Supplementary material for: Evaluation of the Antimicrobial Potential and Characterization of Novel T7-Like Erwinia Bacteriophages
Source: Biology (Basel). 2023 Jan 23;12(2):180. doi: 10.3390/biology12020180 (PMC9953017; doi:10.3390/biology12020180)
Supplement: Supplementary file 1 [file biology-12-00180-s001.zip › Table S1.pdf]

Table S1. Host range of phage pEp_SNUABM_03, pEp_SNUABM_04, pEp_SNUABM_11, pEp_SNUABM_12, and Cocktail phage (mixed pEp_SNUABM_03, 04, 11, 12) against *Erwinia amlyrovora* and *Erwinia pyrifoliae* strains used in this study.

species	strain	isolated		Phage infectivity				
		year	province	pEp_3	pEp_4	pEp_11	pEp_12	cocktail
Erwinia amlyrovora	YKB 14715	2019	Chungcheongbuk	+	+	+	-	+
	YKB 14740	2019	Chungcheongbuk	+	+	-	-	+
	YKB 14742	2019	Chungcheongbuk	+	+	-	-	+
	YKB 14748	2019	Chungcheongbuk	+	+	+	-	+
	YKB 14750	2019	Chungcheongbuk	+	+	+	-	+
	YKB 14754	2019	Chungcheongbuk	+	+	+	-	+
	YKB 14756	2019	Chungcheongbuk	+	+	+	-	+
	YKB 14758	2019	Chungcheongbuk	+	+	+	-	+
	YKB 14768	2019	Chungcheongbuk	+	+	+	-	+
	YKB 14770	2019	Chungcheongbuk	+	+	+	-	+

	YKB 14776	2019	Chungcheongbuk	+	+	+	-	+
	YKB 14778	2019	Chungcheongbuk	+	+	-	-	+
	YKB 14787	2019	Chungcheongnam	+	+	+	-	+
	YKB 14806	2019	Gyeonggi	+	+	-	-	+
	YKB 14808	2019	Gyeonggi	+	+	+	-	+
	YKB 14814	2019	Chungcheongbuk	+	+	+	-	+
	YKB 14818	2019	Chungcheongbuk	+	+	+	-	+
	YKB 14820	2019	Chungcheongbuk	+	+	+	-	+
	YKB 14822	2019	Chungcheongbuk	+	+	-	-	+
	RA0023	2020	Gyeonggi	+	+	+	-	+
	RA0024	2020	Gyeonggi	+	+	-	-	+
	RA0025	2020	Gyeonggi	+	+	+	-	+
	RA0026	2020	Gyeonggi	+	+	+	-	+
	RA0027	2020	Gyeonggi	+	+	+	-	+
	RA0028	2020	Gyeonggi	+	-	-	-	+

RA0029	2020	Gyeonggi	+	+	+	-	+
RA0030	2020	Gyeonggi	+	+	+	+	+
RA0031	2020	Gyeonggi	+	+	+	-	+
RA0032	2020	Gyeonggi	+	+	+	-	+
RA0033	2020	Gyeonggi	-	-	-	-	-
RA0034	2020	Gyeonggi	+	+	+	-	+
RA0035	2020	Gyeonggi	+	+	+	-	+
RA0036	2020	Gyeonggi	+	+	+	-	+
RA0037	2020	Gyeonggi	+	+	+	-	+
RA0038	2020	Jeollabuk	+	+	+	-	+
RA0039	2020	Chungcheongnam	+	+	+	-	+
RA0040	2019	Chungcheongnam	+	+	+	-	+
RA0041	2019	Chungcheongnam	+	+	+	-	+
RA0042	2020	Chungcheongnam	+	+	+	-	+
RA0043	2020	Chungcheongnam	+	+	+	-	+

	RA0044	2020	Chungcheongnam	+	+	+	-	+
	RA0045	2020	Chungcheongbuk	+	+	+	-	+
	RA0046	2020	Chungcheongbuk	+	+	+	-	+
	RA0047	2020	Chungcheongbuk	+	+	+	-	+
	RA0048	2020	Chungcheongbuk	+	+	+	-	+
	RA0049	2020	Chungcheongbuk	+	+	+	-	+
	RA0050	2020	Chungcheongbuk	+	+	+	-	+
	RA0051	2020	Chungcheongbuk	+	+	+	-	+
	RA0052	2020	Chungcheongbuk	+	+	+	-	+
	RA0053	2020	Chungcheongbuk	+	+	+	-	+
	RA0054	2020	Chungcheongbuk	+	+	+	-	+
	RA0055	2020	Chungcheongbuk	+	+	+	-	+
	RA0056	2020	Chungcheongbuk	+	+	+	-	+
	RA0057	2020	Chungcheongbuk	+	+	-	-	+
	RA0058	2020	Chungcheongbuk	+	+	+	-	+

	RA0059	2020	Chungcheongbuk	+	+	+	-	+
	RA0060	2020	Chungcheongbuk	+	+	+	-	+
	RA0061	2020	Chungcheongbuk	+	+	+	-	+
	RA0062	2020	Chungcheongbuk	+	+	+	-	+
	RA0063	2020	Chungcheongbuk	+	+	+	-	+
	RA0064	2020	Chungcheongbuk	+	+	-	-	+
	RA0065	2020	Chungcheongbuk	+	+	+	-	+
	RA0066	2020	Chungcheongbuk	+	+	+	-	+
	RA0067	2020	Chungcheongbuk	+	+	-	-	+
	RA0068	2020	Chungcheongbuk	+	+	+	-	+
	RA0069	2020	Chungcheongbuk	+	+	+	-	+
	RA0070	2020	Chungcheongbuk	+	+	+	-	+
	RA0071	2020	Chungcheongbuk	+	+	+	-	+
	RA0072	2020	Chungcheongbuk	+	+	+	-	+
	RA0073	2020	Chungcheongbuk	+	+	+	-	+

	RA0074	2020	Chungcheongbuk	+	+	+	-	+
	RA0075	2020	Chungcheongbuk	+	+	+	-	+
	RA0076	2020	Chungcheongbuk	+	+	+	-	+
	RA0077	2020	Chungcheongbuk	+	+	+	-	+
	RA0078	2020	Chungcheongbuk	+	+	+	-	+
	RA0079	2020	Chungcheongbuk	+	+	-	-	+
	RA0080	2020	Chungcheongbuk	+	+	+	-	+
	RA0081	2020	Chungcheongbuk	+	+	+	-	+
	RA0082	2020	Chungcheongbuk	+	+	-	-	+
	RA0083	2020	Chungcheongbuk	+	+	-	-	+
	RA0084	2020	Chungcheongbuk	+	+	+	-	+
	RA0085	2020	Chungcheongbuk	+	+	-	-	+
	RA0086	2020	Chungcheongbuk	+	+	-	-	+
	RA0087	2020	Chungcheongbuk	+	+	-	-	+
	RA0088	2020	Chungcheongbuk	+	+	-	-	+

	RA0089	2020	Chungcheongbuk	+	+	+	-	+
	RA0090	2020	Chungcheongbuk	+	+	+	-	+
	RA0091	2020	Chungcheongbuk	+	+	-	-	+
	RA0092	2020	Chungcheongbuk	+	+	-	-	+
	RA0093	2020	Chungcheongbuk	+	+	+	+	+
	RA0094	2020	Chungcheongbuk	+	+	-	-	+
	RA0095	2020	Chungcheongbuk	+	+	+	-	+
Erwinia pyrifoliae	RP0098	2020	Gangwon	-	+	-	+	+
	RP0099	2020	Gangwon	+	+	-	+	+
	RP0100	2020	Gangwon	+	+	+	+	+
	RP0101	2020	Gangwon	+	+	+	+	+
	RP0102	2020	Gangwon	+	+	+	+	+
	RP0103	2020	Gangwon	+	+	+	+	+
	RP0104	2020	Gangwon	+	+	-	+	+
	RP0105	2020	Gangwon	+	+	+	+	+

	RP0106	2020	Gangwon	+	+	+	+	+
	RP0107	2020	Gangwon	+	+	+	+	+
	RP0108	2020	Gangwon	+	+	+	+	+
	RP0109	2020	Gangwon	+	+	+	+	+
	RP0110	2020	Gangwon	-	-	-	-	+
	RP0111	2020	Gyeonggi	+	+	+	+	+
	RP0112	2020	Gyeonggi	+	+	+	+	+
	RP0113	2020	Gyeonggi	+	+	+	+	+
	RP0114	2020	Gyeongsangbuk	+	+	+	+	+
	RP0115	2020	Gyeongsangbuk	+	+	+	+	+
	RP0116	2020	Chungcheongbuk	+	+	+	+	+
	RP0117	2020	Chungcheongbuk	+	+	+	+	+
	RP0118	2020	Chungcheongbuk	+	+	+	+	+
	RP0119	2020	Chungcheongbuk	+	+	+	+	+
	RP0120	2020	Gangwon	+	+	+	+	+

RP0121	2020	Chungcheongbuk	+	+	+	+	+
Total		*E. amylovora*	91 (98.91%)	90 (97.83%)	70 (76.09%)	2 (2.17%)	91 (98.91%)
		E. pyrifoliae	22 (91.67%)	23 (95.83%)	19 (79.17%)	23 (95.83%)	24 (100.00%)
