# Supplementary material for: Evaluation of the Antimicrobial Potential and Characterization of Novel T7-Like Erwinia Bacteriophages
Source: Biology (Basel). 2023 Jan 23;12(2):180. doi: 10.3390/biology12020180 (PMC9953017; doi:10.3390/biology12020180)
Supplement: Supplementary file 1 [file biology-12-00180-s001.zip › Table S2.pdf]

Table S2. Nucleotide identity (%) among the closely related phages. The identity was determined using nucleotide blast algorithm.

	**pEp_03**	**pEp_04**	**pEp_11**	**L1**	**pEp_12**	**Ninurta**
**pEp_03**	100	98.6	98.50	74.53	72.13	72.29
**pEp_04**	-	100	98.18	70.30	72.26	71.83
**pEp_11**	-	-	100	70.78	72.32	71.83
**L1**	-	-	-	100	70.85	71.02
**pEp_12**	-	-	-	-	100	94.66
**Ninurta**	-	-	-	-	-	100
