# Supplementary material for: Evaluation of the Antimicrobial Potential and Characterization of Novel T7-Like Erwinia Bacteriophages
Source: Biology (Basel). 2023 Jan 23;12(2):180. doi: 10.3390/biology12020180 (PMC9953017; doi:10.3390/biology12020180)
Supplement: Supplementary file 1 [file biology-12-00180-s001.zip › Table S3.pdf]

Table S3. Core genes shared by the *Erwinia* phages analyzed in this study.

Erwinia phage			
**pEp_SNUABM_03**	**pEp_SNUABM_04**	**pEp_SNUABM_11**	**pEp_SNUABM_12**
hypothetical protein (QOC57603.1)	hypothetical protein (QOC57658.1)	hypothetical protein (QOC57761.1)	hypothetical protein (QOC57812.1)
putative terminase large subunit (QOC57604.1)	putative terminase large subunit (QOC57659.1)	putative terminase large subunit (QOC57762.1)	putative terminase large subunit (QOC57811.1)
hypothetical protein (QOC57605.1)	hypothetical protein (QOC57660.1)	hypothetical protein (QOC57763.1)	hypothetical protein (QOC57810.1)
putative spanin inner membrane subunit (QOC57606.1)	putative spanin inner membrane subunit (QOC57661.1)	putative spanin inner membrane subunit (QOC57764.1)	putative endopeptidase (QOC57809.1)
putative terminase small subunit (QOC57607.1)	putative terminase small subunit (QOC57662.1)	putative terminase small subunit (QOC57765.1)	putative terminase small subunit (QOC57808.1)
putative type II holin (QOC57608.1)	putative type II holin (QOC57663.1)	putative type II holin (QOC57766.1)	putative type II holin (QOC57807.1)
hypothetical protein (QOC57609.1)	hypothetical protein (QOC57664.1)	hypothetical protein (QOC57767.1)	hypothetical protein (QOC57806.1)
putative tail fiber protein (QOC57610.1)	putative tail fiber protein (QOC57665.1)	putative tail fiber protein (QOC57768.1)	putative tail fiber protein (QOC57855.1)
putative internal virion protein D (QOC57611.1)	putative internal virion protein D (QOC57666.1)	putative internal virion protein D (QOC57769.1)	putative internal virion protein D (QOC57854.1)
putative internal virion protein C (QOC57612.1)	putative internal virion protein C (QOC57667.1)	Internal virion protein C (QOC57770.1)	putative internal virion protein C (QOC57853.1)

putative internal virion protein B (QOC57613.1)	putative internal virion protein B (QOC57668.1)	Internal virion protein C (QOC57771.1)	putative tail protein (QOC57852.1)
putative internal core protein (QOC57614.1)	putative internal core protein (QOC57669.1)	putative internal core protein (QOC57772.1)	internal virion protein A (QOC57851.1)
putative tail tubular protein B (QOC57615.1)	putative tail tubular protein B (QOC57670.1)	putative tail tubular protein B (QOC57773.1)	putative tail tubular protein B (QOC57850.1)
putative tail tubular protein A (QOC57616.1)	putative tail tubular protein A (QOC57671.1)	putative tail tubular protein A (QOC57774.1)	putative tail tubular protein A (QOC57849.1)
putative minor capsid protein (QOC57617.1)	putative minor capsid protein (QOC57672.1)	hypothetical protein (QOC57775.1)	
putative major capsid protein (QOC57618.1)	putative major capsid protein (QOC57673.1)	putative major capsid protein (QOC57776.1)	putative major capsid protein (QOC57847.1)
putative capsid assembly scaffolding protein (QOC57619.1)	putative capsid assembly scaffolding protein (QOC57674.1)	putative capsid assembly scaffolding protein (QOC57777.1)	putative capsid assembly scaffolding protein (QOC57846.1)
putative head to tail connecting protein (QOC57620.1)	putative head to tail connecting protein (QOC57675.1)	putative head to tail connecting protein (QOC57778.1)	putative head to tail joining protein (QOC57845.1)
putative virion assembly protein (QOC57621.1)	putative virion assembly protein (QOC57676.1)	putative virion assembly protein (QOC57779.1)	putative tail assembly protein (QOC57844.1)
hypothetical protein (QOC57622.1)	hypothetical protein (QOC57677.1)	hypothetical protein (QOC57780.1)	hypothetical protein (QOC57843.1)
hypothetical protein (QOC57623.1)	hypothetical protein (QOC57678.1)	hypothetical protein (QOC57781.1)	hypothetical protein (QOC57842.1)
hypothetical protein	hypothetical protein	hypothetical protein	hypothetical protein

(QOC57624.1)	(QOC57679.1)	(QOC57782.1)	(QOC57841.1)
putative exonuclease (QOC57625.1)	putative exonuclease (QOC57680.1)	putative exonuclease (QOC57783.1)	putative exonuclease (QOC57840.1)
hypothetical protein (QOC57626.1)	hypothetical protein (QOC57681.1)	hypothetical protein (QOC57784.1)	hypothetical protein (QOC57839.1)
hypothetical protein (QOC57627.1)	hypothetical protein (QOC57682.1)	hypothetical protein (QOC57785.1)	putative HNS binding protein (QOC57838.1)
putative HNS binding protein (QOC57628.1)	hypothetical protein (QOC57683.1)	putative HNS binding protein (QOC57786.1)	
putative DNA-directed DNA polymerase (QOC57630.1)	putative DNA-directed DNA polymerase (QOC57685.1)	putative DNA-directed DNA polymerase (QOC57685.1)	putative DNA-directed DNA polymerase (QOC57836.1)
putative inhibitor of toxin/antitoxin system (QOC57631.1)	hypothetical protein (QOC57686.1)	putative inhibitor of toxin/antitoxin system (QOC57788.1)	
hypothetical protein (QOC57632.1)	hypothetical protein (QOC57687.1)	hypothetical protein (QOC57789.1)	
hypothetical protein (QOC57633.1)	hypothetical protein (QOC57688.1)	hypothetical protein (QOC57790.1)	
putative DNA helicase (QOC57634.1)	putative DNA helicase (QOC57689.1)	putative DNA helicase (QOC57791.1)	putative DNA helicase (QOC57833.1)

putative N-acetylmuramoyl-L-alanine amidase (QOC57635.1)	putative N-acetylmuramoyl-L-alanine amidase (QOC57690.1)	putative N-acetylmuramoyl-L-alanine amidase (QOC57792.1)	putative N-acetylmuramoyl-L-alanine amidase (QOC57831.1)
putative endonuclease (QOC57636.1)	putative endonuclease (QOC57691.1)	putative endonuclease (QOC57793.1)	putative endonuclease (QOC57830.1)
putative single-stranded DNA-binding protein (QOC57637.1)	putative single-stranded DNA-binding protein (QOC57692.1)	putative single-stranded DNA-binding protein (QOC57794.1)	putative single-stranded DNA-binding protein (QOC57829.1)
putative host RNA polymerase inhibitor (QOC57638.1)	putative host RNA polymerase inhibitor (QOC57693.1)	putative host RNA polymerase inhibitor (QOC57795.1)	putative bacterial RNA polymerase inhibitor (QOC57827.1)
hypothetical protein (QOC57639.1)	hypothetical protein (QOC57694.1)	hypothetical protein (QOC57796.1)	
hypothetical protein (QOC57640.1)	hypothetical protein (QOC57695.1)	hypothetical protein (QOC57797.1)	
hypothetical protein (QOC57641.1)	hypothetical protein (QOC57696.1)	hypothetical protein (QOC57798.1)	
hypothetical protein (QOC57642.1)	hypothetical protein (QOC57697.1)	hypothetical protein (QOC57799.1)	
hypothetical protein (QOC57643.1)	hypothetical protein (QOC57698.1)	hypothetical protein (QOC57800.1)	

putative DNA ligase (QOC57646.1)	putative DNA ligase (QOC57700.1)	putative DNA ligase (QOC57801.1)	putative DNA ligase (QOC57823.1)
putative host dGTPase inhibitor (QOC57647.1)	putative host dGTPase inhibitor (QOC57701.1)	putative host dGTPase inhibitor (QOC57802.1)	putative inhibitor of dGTPase (QOC57822.1)
hypothetical protein (QOC57648.1)	hypothetical protein (QOC57702.1)	hypothetical protein (QOC57803.1)	
hypothetical protein (QOC57649.1)	hypothetical protein (QOC57703.1)	hypothetical protein (QOC57804.1)	hypothetical protein (QOC57820.1)
putative RNA polymerase (QOC57650.1)	putative RNA polymerase (QOC57704.1)	putative RNA polymerase (QOC57805.1)	putative RNA polymerase (QOC57819.1)
hypothetical protein (QOC57652.1)	hypothetical protein (QOC57655.1)	hypothetical protein (QOC57757.1)	hypothetical protein (QOC57817.1)
putative S-adenosyl-L-methionine hydrolase (QOC57654.1)	putative S-adenosyl-L-methionine hydrolase (QOC57657.1)	putative S-adenosyl-L-methionine hydrolase (QOC57759.1)	putative S-adenosyl-L-methionine hydrolase (QOC57813.1)
