# Supplementary material for: Evaluation of the Antimicrobial Potential and Characterization of Novel T7-Like Erwinia Bacteriophages
Source: Biology (Basel). 2023 Jan 23;12(2):180. doi: 10.3390/biology12020180 (PMC9953017; doi:10.3390/biology12020180)
Supplement: Supplementary file 1 [file biology-12-00180-s001.zip › Table S4.pdf]

Table S4. Functional categories of the predicted open reading frames (ORFs) in *Erwinia* phage pEp_SNUABM_03.

Group	Locus tag	Encoded protein	Related organism	Query cover (%)	Identity (%)
Hypothetical protein	pEp_SNUABM_03_00001	Hypothetical protein	*Erwinia* phage vB_EamP-L1	96	60.42
Structure and packaging	pEp_SNUABM_03_00002	putative terminase large subunit	*Erwinia* phage pEp_SNUABM_09	100	99.83
Hypothetical protein	pEp_SNUABM_03_00003	Hypothetical protein	*Erwinia* phage pEp_SNUABM_09	100	96.57
Lysis	pEp_SNUABM_03_00004	putative spanin inner membrane subunit	*Erwinia* phage pEp_SNUABM_09	100	100
Structure and packaging	pEp_SNUABM_03_00005	putative terminase small subunit	*Erwinia* phage pEp_SNUABM_09	100	98.85
Lysis	pEp_SNUABM_03_00006	putative type II holin	*Erwinia* phage pEp_SNUABM_09	100	100
Hypothetical protein	pEp_SNUABM_03_00007	Hypothetical protein	*Erwinia* phage pEp_SNUABM_09	100	98.45
Structure and packaging	pEp_SNUABM_03_00008	putative tail fiber protein	*Erwinia* phage pEp_SNUABM_09	100	99.43
Structure and packaging	pEp_SNUABM_03_00009	putative internal virion protein D	*Erwinia* phage pEp_SNUABM_09	100	99.24
Structure and packaging	pEp_SNUABM_03_00010	putative internal virion protein C	*Erwinia* phage pEp_SNUABM_09	10	100

Structure and packaging	pEp_SNUABM_03_00011	putative internal virion protein B,	*Erwinia* phage pEp_SNUABM_09	100	100
Structure and packaging	pEp_SNUABM_03_00012	putative internal core protein	*Erwinia* phage pEp_SNUABM_09	100	98.62
Structure and packaging	pEp_SNUABM_03_00013	putative tail tubular protein B	*Erwinia* phage pEp_SNUABM_09	100	99.62
Structure and packaging	pEp_SNUABM_03_00014	putative tail tubular protein A	*Erwinia* phage pEp_SNUABM_09	100	100
Structure and packaging	pEp_SNUABM_03_00015	putative minor capsid protein	*Erwinia* phage pEp_SNUABM_09	100	95
Structure and packaging	pEp_SNUABM_03_00016	putative major capsid protein	*Erwinia* phage pEp_SNUABM_09	100	100
Structure and packaging	pEp_SNUABM_03_00017	putative capsid assembly scaffolding protein	*Erwinia* phage pEp_SNUABM_09	100	99.36
Structure and packaging	pEp_SNUABM_03_00018	putative head to tail connecting protein	*Erwinia* phage pEp_SNUABM_09	100	100
Structure and packaging	pEp_SNUABM_03_00019	putative virion assembly protein	*Erwinia* phage pEp_SNUABM_09	100	100
Hypothetical protein	pEp_SNUABM_03_00020	Hypothetical protein	*Erwinia* phage pEp_SNUABM_09	100	100
Hypothetical protein	pEp_SNUABM_03_00021	Hypothetical protein	*Erwinia* phage pEp_SNUABM_09	100	98.77

Hypothetical protein	pEp_SNUABM_03_00022	Hypothetical protein	*Erwinia* phage pEp_SNUABM_09	100	97.5
Nucleotide regulation	pEp_SNUABM_03_00023	putative exonuclease	*Erwinia* phage pEp_SNUABM_09	100	99.67
Hypothetical protein	pEp_SNUABM_03_00024	Hypothetical protein	*Erwinia* phage pEp_SNUABM_09	100	100
Hypothetical protein	pEp_SNUABM_03_00025	Hypothetical protein	*Erwinia* phage pEp_SNUABM_09	100	100
Nucleotide regulation	pEp_SNUABM_03_00026	putative HNS binding protein	*Erwinia* phage pEp_SNUABM_09	100	97.8
Hypothetical protein	pEp_SNUABM_03_00027	Hypothetical protein	*Erwinia* phage pEp_SNUABM_09	100	100
Nucleotide regulation	pEp_SNUABM_03_00028	putative DNA-directed DNA polymerase	*Erwinia* phage pEp_SNUABM_09	100	99.72
Additional function	pEp_SNUABM_03_00029	putative inhibitor of toxin/antitoxin system	*Erwinia* phage pEp_SNUABM_09	100	89.47
Hypothetical protein	pEp_SNUABM_03_00030	Hypothetical protein	*Erwinia* phage pEp_SNUABM_09	100	100
Hypothetical protein	pEp_SNUABM_03_00031	Hypothetical protein	N/A[a]	N/A	N/A

Nucleotide regulation	pEp_SNUABM_03_00032	putative DNA helicase	*Erwinia* phage pEp_SNUABM_09	89	100
Lysis	pEp_SNUABM_03_00033	putative N-acetylmuramoyl-L-alanine amidase	*Erwinia* phage pEp_SNUABM_09	100	98.68
Nucleotide regulation	pEp_SNUABM_03_00034	putative endonuclease	*Erwinia* phage pEp_SNUABM_09	100	100
Nucleotide regulation	pEp_SNUABM_03_00035	putative single-stranded DNA-binding protein	*Erwinia* phage pEp_SNUABM_09	100	99.13
Additional function	pEp_SNUABM_03_00036	putative host RNA polymerase inhibitor	*Erwinia* phage pEp_SNUABM_09	100	100
Hypothetical protein	pEp_SNUABM_03_00037	Hypothetical protein	*Erwinia* phage pEp_SNUABM_09	100	100
Hypothetical protein	pEp_SNUABM_03_00038	Hypothetical protein	*Erwinia* phage pEp_SNUABM_09	98	85.88
Hypothetical protein	pEp_SNUABM_03_00039	Hypothetical protein	*Erwinia* phage pEp_SNUABM_09	100	100
Hypothetical protein	pEp_SNUABM_03_00040	Hypothetical protein	*Erwinia* phage pEp_SNUABM_09	100	100
Hypothetical protein	pEp_SNUABM_03_00041	Hypothetical protein	*Erwinia* phage pEp_SNUABM_09	100	98.21

Hypothetical protein	pEp_SNUABM_03_00042	Hypothetical protein	*Erwinia* phage pEp_SNUABM_09	100	88.52
Hypothetical protein	pEp_SNUABM_03_00043	Hypothetical protein	*Erwinia* phage pEp_SNUABM_09	100	98.36
Nucleotide regulation	pEp_SNUABM_03_00044	putative DNA ligase	*Erwinia* phage pEp_SNUABM_09	100	93.24
Nucleotide regulation	pEp_SNUABM_03_00045	putative host dGTPase inhibitor	*Erwinia* phage pEp_SNUABM_09	62	98.08
Hypothetical protein	pEp_SNUABM_03_00046	Hypothetical protein	*Erwinia* phage pEp_SNUABM_09	100	100
Hypothetical protein	pEp_SNUABM_03_00047	Hypothetical protein	*Erwinia* phage pEp_SNUABM_09	100	96.37
Nucleotide regulation	pEp_SNUABM_03_00048	putative RNA polymerase	*Erwinia* phage pEp_SNUABM_09	100	100
Nucleotide regulation	pEp_SNUABM_03_00049	putative protein kinase	*Dickeya* phage Ninurta	70	52.87
Hypothetical protein	pEp_SNUABM_03_00050	Hypothetical protein	*Erwinia* phage pEp_SNUABM_09	79	100
Hypothetical protein	pEp_SNUABM_03_00051	Hypothetical protein	N/A	N/A	N/A
Nucleotide regulation	pEp_SNUABM_03_00052	putative S-adenosyl-L-methionine hydrolase	*Erwinia* phage pEp_SNUABM_09	95	95.12

[a]N/A, Not available.
