# Supplementary material for: Evaluation of the Antimicrobial Potential and Characterization of Novel T7-Like Erwinia Bacteriophages
Source: Biology (Basel). 2023 Jan 23;12(2):180. doi: 10.3390/biology12020180 (PMC9953017; doi:10.3390/biology12020180)
Supplement: Supplementary file 1 [file biology-12-00180-s001.zip › Table S5.pdf]

Table S5. Functional categories of the predicted open reading frames (ORFs) in *Erwinia* phage pEp_SNUABM_04.

Group	Locus tag	Encoded protein	Related organism	Query cover (%)	Identity (%)
Hypothetical protein	pEp_SNUABM_04_00001	Hypothetical protein	*Erwinia* phage pEp_SNUABM_09	78	98.04
Hypothetical protein	pEp_SNUABM_04_00002	Hypothetical protein	*Erwinia* phage pEp_SNUABM_09	97	100
Nucleotide regulation	pEp_SNUABM_04_00003	putative S-adenosyl-L-methionine hydrolase	*Erwinia* phage pEp_SNUABM_09	98	99.35
Hypothetical protein	pEp_SNUABM_04_00004	Hypothetical protein	*Yersinia* phage Berlin	94	54.17
Structure and packaging	pEp_SNUABM_04_00005	putative terminase large subunit	*Erwinia* phage pEp_SNUABM_09	99	99.83
Hypothetical protein	pEp_SNUABM_04_00006	Hypothetical protein	*Erwinia* phage pEp_SNUABM_09	100	95.59
Lysis	pEp_SNUABM_04_00007	putative spanin inner membrane subunit	*Erwinia* phage pEp_SNUABM_09	100	99.32
Nucleotide regulation	pEp_SNUABM_04_00008	putative terminase small subunit	*Erwinia* phage pEp_SNUABM_09	100	100
Lysis	pEp_SNUABM_04_00009	putative type II holin	*Erwinia* phage pEp_SNUABM_09	100	100
Hypothetical protein	pEp_SNUABM_04_00010	Hypothetical protein	*Erwinia* phage pEp_SNUABM_09	100	97.67

Structure and packaging	pEp_SNUABM_04_00011	putative tail fiber protein	*Erwinia* phage pEp_SNUABM_09	100	99.43
Structure and packaging	pEp_SNUABM_04_00012	putative internal virion protein D	*Erwinia* phage pEp_SNUABM_09	100	99.24
Structure and packaging	pEp_SNUABM_04_00013	putative internal virion protein C	*Erwinia* phage pEp_SNUABM_09	100	99.87
Structure and packaging	pEp_SNUABM_04_00014	putative internal virion protein B	*Erwinia* phage pEp_SNUABM_09	100	100
Structure and packaging	pEp_SNUABM_04_00015	putative internal core protein	*Erwinia* phage pEp_SNUABM_09	100	97.93
Structure and packaging	pEp_SNUABM_04_00016	putative tail tubular protein B	*Erwinia* phage pEp_SNUABM_09	100	99.75
Structure and packaging	pEp_SNUABM_04_00017	putative tail tubular protein A	*Erwinia* phage pEp_SNUABM_09	100	100
Structure and packaging	pEp_SNUABM_04_00018	putative minor capsid protein	*Erwinia* phage pEp_SNUABM_09	100	97.5
Structure and packaging	pEp_SNUABM_04_00019	putative major capsid protein	*Erwinia* phage pEp_SNUABM_09	100	100
Structure and packaging	pEp_SNUABM_04_00020	putative capsid assembly scaffolding protein	*Erwinia* phage pEp_SNUABM_09	100	99.36
Structure and packaging	pEp_SNUABM_04_00021	putative head to tail connecting protein	*Erwinia* phage pEp_SNUABM_09	100	100
Structure and packaging	pEp_SNUABM_04_00022	putative virion assembly protein	*Erwinia* phage pEp_SNUABM_09	100	100
Hypothetical protein	pEp_SNUABM_04_00023	Hypothetical protein	*Erwinia* phage pEp_SNUABM_09	100	100

Hypothetical protein	pEp_SNUABM_04_00024	Hypothetical protein	*Erwinia* phage pEp_SNUABM_09	100	98.77
Hypothetical protein	pEp_SNUABM_04_00025	Hypothetical protein	*Erwinia* phage pEp_SNUABM_09	100	97.5
Nucleotide regulation	pEp_SNUABM_04_00026	putative exonuclease	*Erwinia* phage pEp_SNUABM_09	100	99.34
Hypothetical protein	pEp_SNUABM_04_00027	Hypothetical protein	*Erwinia* phage pEp_SNUABM_09	100	98.98
Hypothetical protein	pEp_SNUABM_04_00028	Hypothetical protein	*Erwinia* phage pEp_SNUABM_09	100	100
Hypothetical protein	pEp_SNUABM_04_00029	Hypothetical protein	*Erwinia* phage pEp_SNUABM_09	100	98.9
Hypothetical protein	pEp_SNUABM_04_00030	Hypothetical protein	*Erwinia* phage pEp_SNUABM_09	100	98.11
Nucleotide regulation	pEp_SNUABM_04_00031	putative DNA-directed DNA polymerase	*Erwinia* phage pEp_SNUABM_09	100	100
Hypothetical protein	pEp_SNUABM_04_00032	Hypothetical protein	*Erwinia* phage pEp_SNUABM_09	100	89.47
Hypothetical protein	pEp_SNUABM_04_00033	Hypothetical protein	*Erwinia* phage pEp_SNUABM_09	100	97.14
Hypothetical protein	pEp_SNUABM_04_00034	Hypothetical protein	N/A[a]	N/A	N/A
Nucleotide regulation	pEp_SNUABM_04_00035	putative DNA helicase	*Erwinia* phage pEp_SNUABM_09	89	100
Lysis	pEp_SNUABM_04_00036	putative N-acetylmuramoyl-L-alanine amidase	*Erwinia* phage pEp_SNUABM_09	100	98.68

Nucleotide regulation	pEp_SNUABM_04_00037	putative endonuclease	*Erwinia* phage pEp_SNUABM_09	100	100
Nucleotide regulation	pEp_SNUABM_04_00038	putative single-stranded DNA-binding protein	*Erwinia* phage pEp_SNUABM_09	100	99.13
Additional function	pEp_SNUABM_04_00039	putative host RNA polymerase inhibitor	*Erwinia* phage pEp_SNUABM_09	100	100
Hypothetical protein	pEp_SNUABM_04_00040	Hypothetical protein	*Erwinia* phage pEp_SNUABM_09	100	100
Hypothetical protein	pEp_SNUABM_04_00041	Hypothetical protein	*Erwinia* phage pEp_SNUABM_09	99	61.78
Hypothetical protein	pEp_SNUABM_04_00042	Hypothetical protein	*Erwinia* phage pEp_SNUABM_09	100	98.88
Hypothetical protein	pEp_SNUABM_04_00043	Hypothetical protein	*Erwinia* phage pEp_SNUABM_09	100	100
Hypothetical protein	pEp_SNUABM_04_00044	Hypothetical protein	*Erwinia* phage pEp_SNUABM_09	100	98.21
Hypothetical protein	pEp_SNUABM_04_00045	Hypothetical protein	*Erwinia* phage pEp_SNUABM_09	100	88.52
Nucleotide regulation	pEp_SNUABM_04_00046	putative DNA ligase	*Erwinia* phage pEp_SNUABM_09	100	91.6
Additional function	pEp_SNUABM_04_00047	putative host dGTPase inhibitor	*Erwinia* phage pEp_SNUABM_09	62	98.08
Hypothetical protein	pEp_SNUABM_04_00048	Hypothetical protein	*Erwinia* phage pEp_SNUABM_09	100	100
Hypothetical protein	pEp_SNUABM_04_00049	Hypothetical protein	*Erwinia* phage pEp_SNUABM_09	100	98.45

Structure and packaging	pEp_SNUABM_04_00050	putative RNA polymerase	*Erwinia* phage pEp_SNUABM_09	100	100

[a]N/A, Not available
