# Supplementary material for: Evaluation of the Antimicrobial Potential and Characterization of Novel T7-Like Erwinia Bacteriophages
Source: Biology (Basel). 2023 Jan 23;12(2):180. doi: 10.3390/biology12020180 (PMC9953017; doi:10.3390/biology12020180)
Supplement: Supplementary file 1 [file biology-12-00180-s001.zip › Table S6.pdf]

Table S6. Functional categories of the predicted open reading frames (ORFs) in *Erwinia* phage pEp_SNUABM_11.

Group	Locus tag	Encoded protein	Related organism	Query cover (%)	Identity (%)
Hypothetical protein	pEp_SNUABM_11_00001	Hypothetical protein	*Erwinia* phage pEp_SNUABM_09	79	100
Hypothetical protein	pEp_SNUABM_11_00002	Hypothetical protein	*Erwinia* phage pEp_SNUABM_09	100	97.73
Nucleotide regulation	pEp_SNUABM_11_00003	putative S-adenosyl-L-methionine hydrolase	*Erwinia* phage pEp_SNUABM_09	99	98.05
Hypothetical protein	pEp_SNUABM_11_00004	Hypothetical protein	*Yersinia* phage Berlin	45	59.38
Hypothetical protein	pEp_SNUABM_11_00005	Hypothetical protein	*Erwinia* phage vB_EamP-L1	96	60.42
Structure and packaging	pEp_SNUABM_11_00006	putative terminase large subunit	*Erwinia* phage pEp_SNUABM_09	100	99.83
Hypothetical protein	pEp_SNUABM_11_00007	Hypothetical protein	*Erwinia* phage pEp_SNUABM_09	100	94.12
Lysis	pEp_SNUABM_11_00008	putative spanin inner membrane subunit	*Erwinia* phage pEp_SNUABM_09	100	100
Structure and packaging	pEp_SNUABM_11_00009	putative terminase small subunit	*Erwinia* phage pEp_SNUABM_09	100	100
Lysis	pEp_SNUABM_11_00010	putative type II holin	*Erwinia* phage pEp_SNUABM_09	100	100

Hypothetical protein	pEp_SNUABM_11_00011	Hypothetical protein	*Erwinia* phage pEp_SNUABM_09	100	98.45
Structure and packaging	pEp_SNUABM_11_00012	putative tail fiber protein	*Erwinia* phage pEp_SNUABM_09	100	99.06
Structure and packaging	pEp_SNUABM_11_00013	putative internal virion protein D	*Erwinia* phage pEp_SNUABM_09	100	99.01
Structure and packaging	pEp_SNUABM_11_00014	Internal virion protein C	*Erwinia* phage pEp_SNUABM_09	100	99.87
Structure and packaging	pEp_SNUABM_11_00015	Internal virion protein C	*Erwinia* phage pEp_SNUABM_09	100	100
Structure and packaging	pEp_SNUABM_11_00016	putative internal core protein	*Erwinia* phage pEp_SNUABM_09	100	97.24
Structure and packaging	pEp_SNUABM_11_00017	putative tail tubular protein B	*Erwinia* phage pEp_SNUABM_09	100	99.87
Structure and packaging	pEp_SNUABM_11_00018	putative tail tubular protein A	*Erwinia* phage pEp_SNUABM_09	100	100
Hypothetical protein	pEp_SNUABM_11_00019	Hypothetical protein	*Erwinia* phage pEp_SNUABM_09	100	95
Structure and packaging	pEp_SNUABM_11_00020	putative major capsid protein	*Erwinia* phage pEp_SNUABM_09	100	99.71
Structure and packaging	pEp_SNUABM_11_00021	putative capsid assembly scaffolding protein	*Erwinia* phage pEp_SNUABM_09	100	99.36
Structure and packaging	pEp_SNUABM_11_00022	putative head to tail connecting protein	*Erwinia* phage pEp_SNUABM_09	100	100
Structure and packaging	pEp_SNUABM_11_00023	putative virion assembly protein	*Erwinia* phage pEp_SNUABM_09	100	100

Hypothetical protein	pEp_SNUABM_11_00024	Hypothetical protein	*Erwinia* phage pEp_SNUABM_09	100	100
Hypothetical protein	pEp_SNUABM_11_00025	Hypothetical protein	*Erwinia* phage pEp_SNUABM_09	100	98.77
Hypothetical protein	pEp_SNUABM_11_00026	Hypothetical protein	*Erwinia* phage pEp_SNUABM_09	100	95
Nucleotide regulation	pEp_SNUABM_11_00027	putative exonuclease	*Erwinia* phage pEp_SNUABM_09	100	99.67
Hypothetical protein	pEp_SNUABM_11_00028	Hypothetical protein	*Erwinia* phage pEp_SNUABM_09	100	100
Hypothetical protein	pEp_SNUABM_11_00029	Hypothetical protein	*Erwinia* phage pEp_SNUABM_09	100	100
Nucleotide regulation	pEp_SNUABM_11_00030	putative HNS binding protein	*Erwinia* phage pEp_SNUABM_09	100	98.9
Nucleotide regulation	pEp_SNUABM_11_00031	putative DNA-directed DNA polymerase	*Erwinia* phage pEp_SNUABM_09	100	99.86
Additional function	pEp_SNUABM_11_00032	putative inhibitor of toxin/antitoxin system	*Erwinia* phage pEp_SNUABM_09	100	90.53
Hypothetical protein	pEp_SNUABM_11_00033	Hypothetical protein	*Erwinia* phage pEp_SNUABM_09	100	100
Hypothetical protein	pEp_SNUABM_11_00034	Hypothetical protein	N/A[a]	N/A	N/A
Nucleotide regulation	pEp_SNUABM_11_00035	putative DNA helicase	*Erwinia* phage pEp_SNUABM_09	89	99.8

Lysis	pEp_SNUABM_11_00036	putative N-acetylmuramoyl-L-alanine amidase	*Erwinia* phage pEp_SNUABM_09	100	99.34
Nucleotide regulation	pEp_SNUABM_11_00037	putative endonuclease	*Erwinia* phage pEp_SNUABM_09	100	100
Nucleotide regulation	pEp_SNUABM_11_00038	putative single-stranded DNA-binding protein	*Erwinia* phage pEp_SNUABM_09	100	98.69
Additional function	pEp_SNUABM_11_00039	putative host RNA polymerase inhibitor	*Erwinia* phage pEp_SNUABM_09	100	100
hypothetical protein	pEp_SNUABM_11_00040	hypothetical protein	*Erwinia* phage pEp_SNUABM_09	100	100
Hypothetical protein	pEp_SNUABM_11_00041	Hypothetical protein	*Erwinia* phage pEp_SNUABM_09	100	82.99
Hypothetical protein	pEp_SNUABM_11_00042	Hypothetical protein	*Erwinia* phage pEp_SNUABM_09	100	96.7
Hypothetical protein	pEp_SNUABM_11_00043	Hypothetical protein	*Erwinia* phage pEp_SNUABM_09	100	100
Hypothetical protein	pEp_SNUABM_11_00044	Hypothetical protein	*Erwinia* phage pEp_SNUABM_09	100	98.21
Nucleotide regulation	pEp_SNUABM_11_00045	putative DNA ligase	*Erwinia* phage pEp_SNUABM_09	100	86.53
Additional function	pEp_SNUABM_11_00046	putative host dGTPase inhibitor	*Erwinia* phage pEp_SNUABM_09	62	98.08
Hypothetical protein	pEp_SNUABM_11_00047	Hypothetical protein	*Erwinia* phage pEp_SNUABM_09	100	100

Hypothetical protein	pEp_SNUABM_11_00048	Hypothetical protein	*Erwinia* phage pEp_SNUABM_09	100	95.34
Nucleotide regulation	pEp_SNUABM_11_00049	putative RNA polymerase	*Erwinia* phage pEp_SNUABM_09	100	100

[a]N/A, Not available.
