# Supplementary material for: Evaluation of the Antimicrobial Potential and Characterization of Novel T7-Like Erwinia Bacteriophages
Source: Biology (Basel). 2023 Jan 23;12(2):180. doi: 10.3390/biology12020180 (PMC9953017; doi:10.3390/biology12020180)
Supplement: Supplementary file 1 [file biology-12-00180-s001.zip › Table S7.pdf]

Table S7. Functional categories of the predicted open reading frames (ORFs) in *Erwinia* phage pEp_SNUABM_12.

Group	Locus tag	Encoded protein	Related organism	Query cover (%)	Identity (%)
Hypothetical protein	pEp_SNUABM_12_00001	Hypothetical protein	*Klebsiella* phage vB_KpnP_Sibilus	100	98.41
Lysis	pEp_SNUABM_12_00002	putative type II holin	*Dickeya* phage Ninurta	100	100
Structure and packaging	pEp_SNUABM_12_00003	putative terminase small subunit	*Dickeya* phage Ninurta	100	98.85
Lysis	pEp_SNUABM_12_00004	putative endopeptidase	*Klebsiella* phage vB_KpnP_Sibilus	100	99.38
Hypothetical protein	pEp_SNUABM_12_00005	Hypothetical protein	*Klebsiella* phage vB_KpnP_Sibilus	100	92.2
Structure and packaging	pEp_SNUABM_12_00006	putative terminase large subunit	*Klebsiella* phage vB_KpnP_NahiliMali	100	99.66
Hypothetical protein	pEp_SNUABM_12_00007	Hypothetical protein	*Klebsiella* phage vB_KpnP_NahiliMali	100	98.08
Nucleotide regulation	pEp_SNUABM_12_00008	putative S-adenosyl-L-methionine hydrolase	*Klebsiella* phage vB_KpnP_Sibilus	100	100
Hypothetical protein	pEp_SNUABM_12_00009	Hypothetical protein	*Klebsiella* phage vB_KpnP_Sibilus	100	97.96
Hypothetical protein	pEp_SNUABM_12_00010	Hypothetical protein	*Dickeya* phage vB_DsoP_JA10	97	95.65

Hypothetical protein	pEp_SNUABM_12_00011	Hypothetical protein	*Klebsiella* phage vB_KpnP_Sibilus	100	100
Hypothetical protein	pEp_SNUABM_12_00012	Hypothetical protein	*Klebsiella* phage vB_KpnP_Sibilus	100	100
Nucleotide regulation	pEp_SNUABM_12_00013	putative protein kinase	*Dickeya* phage vB_DsoP_JA10	100	83.38
Nucleotide regulation	pEp_SNUABM_12_00014	putative RNA polymerase	*Dickeya* phage vB_DsoP_JA10	100	99.32
Hypothetical protein	pEp_SNUABM_12_00015	Hypothetical protein	*Dickeya* phage Ninurta	100	98.77
Hypothetical protein	pEp_SNUABM_12_00016	Hypothetical protein	*Klebsiella* phage vB_KpnP_Sibilus	100	98.28
Additional function	pEp_SNUABM_12_00017	putative inhibitor of dGTPase	*Dickeya* phage vB_DsoP_JA10	100	79.31
Nucleotide regulation	pEp_SNUABM_12_00018	putative DNA ligase	*Dickeya* phage vB_DsoP_JA10	99	98.54
Hypothetical protein	pEp_SNUABM_12_00019	Hypothetical protein	*Klebsiella* phage vB_KpnP_Sibilus	100	100
Hypothetical protein	pEp_SNUABM_12_00020	Hypothetical protein	*Dickeya* phage vB_DsoP_JA10	100	98.82
Hypothetical protein	pEp_SNUABM_12_00021	Hypothetical protein	*Dickeya* phage Ninurta	100	98.58
Additional function	pEp_SNUABM_12_00022	putative bacterial RNA polymerase inhibitor	*Klebsiella* phage vB_KpnP_NahiliMali	100	100
Hypothetical protein	pEp_SNUABM_12_00023	Hypothetical protein	*Dickeya* phage vB_DsoP_JA10	100	99.18

Nucleotide regulation	pEp_SNUABM_12_00024	putative single-stranded DNA-binding protein	*Klebsiella* phage vB_KpnP_Sibilus	100	99.57
Nucleotide regulation	pEp_SNUABM_12_00025	putative endonuclease	*Klebsiella* phage vB_KpnP_Sibilus	100	100
Lysis	pEp_SNUABM_12_00026	putative N-acetylmuramoyl-L-alanine amidase	*Klebsiella* phage vB_KpnP_Sibilus	100	98.68
Nucleotide regulation	pEp_SNUABM_12_00027	putative nucleotidyltransferase	*Klebsiella* phage vB_KpnP_Sibilus	100	97.01
Nucleotide regulation	pEp_SNUABM_12_00028	putative DNA helicase	*Klebsiella* phage vB_KpnP_Sibilus	100	99.3
Hypothetical protein	pEp_SNUABM_12_00029	Hypothetical protein	*Dickeya* phage vB_DsoP_JA10	100	93.51
Hypothetical protein	pEp_SNUABM_12_00030	Hypothetical protein	*Klebsiella* phage vB_KpnP_Sibilus	100	100
Nucleotide regulation	pEp_SNUABM_12_00031	putative DNA-directed DNA polymerase	*Dickeya* phage Ninurta	100	99.71
Nucleotide regulation	pEp_SNUABM_12_00032	putative HNS binding protein	*Klebsiella* phage vB_KpnP_Sibilus	100	96.15
Nucleotide regulation	pEp_SNUABM_12_00033	putative HNS binding protein	*Klebsiella* phage vB_KpnP_NahiliMali	100	100
Hypothetical protein	pEp_SNUABM_12_00034	Hypothetical protein	*Klebsiella* phage vB_KpnP_Sibilus	100	98.02
Nucleotide regulation	pEp_SNUABM_12_00035	putative exonuclease	*Klebsiella* phage vB_KpnP_Sibilus	100	97.43

Hypothetical protein	pEp_SNUABM_12_00036	Hypothetical protein	N/A[a]	N/A	N/A
Hypothetical protein	pEp_SNUABM_12_00037	Hypothetical protein	*Dickeya* phage Ninurta	100	98.81
Hypothetical protein	pEp_SNUABM_12_00038	Hypothetical protein	*Klebsiella* phage vB_KpnP_Sibilus	100	98.98
Structure and packaging	pEp_SNUABM_12_00039	putative tail assembly protein	*Klebsiella* phage vB_KpnP_Sibilus	100	98.08
Structure and packaging	pEp_SNUABM_12_00040	putative head to tail joining protein	*Dickeya* phage vB_DsoP_JA10	100	100
Structure and packaging	pEp_SNUABM_12_00041	putative capsid assembly scaffolding protein	*Klebsiella* phage vB_KpnP_Sibilus	100	98.6
Structure and packaging	pEp_SNUABM_12_00042	putative major capsid protein	*Dickeya* phage Ninurta	100	99.71
Structure and packaging	pEp_SNUABM_12_00043	putative minor capsid protein	*Klebsiella* phage vB_KpnP_Sibilus	100	98.68
Structure and packaging	pEp_SNUABM_12_00044	putative tail tubular protein A	*Klebsiella* phage vB_KpnP_Sibilus	100	98.97
Structure and packaging	pEp_SNUABM_12_00045	putative tail tubular protein B	*Klebsiella* phage vB_KpnP_Sibilus	100	99.37

Structure and packaging	pEp_SNUABM_12_00046	internal virion protein A	*Klebsiella* phage vB_KpnP_Sibilus	100	99.3
Structure and packaging	pEp_SNUABM_12_00047	putative tail protein	*Klebsiella* phage vB_KpnP_NahiliMali	100	98.48
Structure and packaging	pEp_SNUABM_12_00048	putative internal virion protein C	*Klebsiella* phage vB_KpnP_Sibilus	100	99.47
Structure and packaging	pEp_SNUABM_12_00049	putative internal virion protein D	*Klebsiella* phage vB_KpnP_Sibilus	100	99.17
Structure and packaging	pEp_SNUABM_12_00050	putative tail fiber protein	*Klebsiella* phage vB_KpnP_Sibilus	100	96.38

[a]N/A, Not available.
